# Lung macrophages drive mucus production and steroid-resistant inflammation in chronic bronchitis

**DOI:** 10.1186/s12931-021-01762-4

**Published:** 2021-06-07

**Authors:** Kristina Andelid, Karolina Öst, Anders Andersson, Esha Mohamed, Zala Jevnikar, Lowie E. G. W. Vanfleteren, Melker Göransson

**Affiliations:** 1 COPD Center, Dept of Respiratory Medicine and Allergology, Sahlgrenska University Hospital, Gothenburg, Sweden; 2 COPD Center, Dept of Internal Medicine and Clinical Nutrition, Institute of Medicine, Sahlgrenska Academy, University of Gothenburg, Gothenburg, Sweden; 3 Department of Bioscience COPD/IPF, Research and Early Development, Respiratory and Immunology (R&I), BioPharmaceuticals R&D, AstraZeneca, Pepparedsleden 1, 43153 Gothenburg, Sweden; 4 Data Sciences and Quantitative Biology, Discovery Sciences, BioPharmaceuticals R&D, AstraZeneca, Gothenburg, Sweden; 5 Translational Science and Experimental Medicine, Research and Early Development, Respiratory and Immunology (R&I), BioPharmaceuticals R&D, AstraZeneca, Gothenburg, Sweden

**Keywords:** COPD, Chronic bronchitis, Sputum macrophages, Steroid resistance, TNFα, MUC5B, Macrophage-epithelial cross-talk

## Abstract

**Background:**

Patients with chronic obstructive pulmonary disease (COPD) frequently suffer from chronic bronchitis (CB) and display steroid-resistant inflammation with increased sputum neutrophils and macrophages. Recently, a causal link between mucus hyper-concentration and disease progression of CB has been suggested.

**Methods:**

In this study, we have evaluated the steroid sensitivity of purified, patient-derived sputum and alveolar macrophages and used a novel mechanistic cross-talk assay to examine how macrophages and bronchial epithelial cells cross-talk to regulate MUC5B production.

**Results:**

We demonstrate that sputum plug macrophages isolated from COPD patients with chronic bronchitis (COPD/CB) are chronically activated and only partially respond to ex vivo corticosteroid treatment compared to alveolar macrophages isolated from lung resections. Further, we show that pseudo-stratified bronchial epithelial cells grown in air–liquid-interface are inert to direct bacterial lipopolysaccharide stimulation and that macrophages are able to relay this signal and activate the CREB/AP-1 transcription factor complex and subsequent MUC5B expression in epithelial cells through a soluble mediator. Using recombinant protein and neutralizing antibodies, we identified a key role for TNFα in this cross-talk.

**Conclusions:**

For the first time, we describe ex vivo pharmacology in purified human sputum macrophages isolated from chronic bronchitis COPD patients and identify a possible basis for the steroid resistance frequently seen in this population. Our data pinpoint a critical role for chronically activated sputum macrophages in perpetuating TNFα-dependent signals driving mucus hyper-production. Targeting the chronically activated mucus plug macrophage phenotype and interfering with aberrant macrophage-epithelial cross-talk may provide a novel strategy to resolve chronic inflammatory lung disease.

**Supplementary Information:**

The online version contains supplementary material available at 10.1186/s12931-021-01762-4.

## Background

Chronic obstructive pulmonary disease (COPD) is now well recognized to be a heterogeneous disease with a spectrum of different phenotypes at the clinical level and endotypes at the biological level. A subset of patients with COPD show increased eosinophils in blood and a favorable response to corticosteroids [1]. However, the most common phenotype of COPD displays an increased number of neutrophils in sputum reflecting a neutrophilic lung disease. This neutrophilic phenotype responds poorly to corticosteroids and there is an urgent need to find new ways to treat this group of patients [2].

Chronic bronchitis (CB) is a constant inflammation of the lining of the bronchial tubes resulting in hypertrophy of the mucus-producing glands found in the mucosa of large cartilaginous airways and epithelial surface goblet cell hyperplasia in the central airways. Among COPD patients, CB occurs in 14–74% of cases, depending on the particular definition used [3].

CB is most commonly defined epidemiologically as cough and sputum production for ≥ 3 months for at least 2 consecutive years [4]. Patients with both COPD and CB exhibit mucin hyper-expression (5) which lead to mucus hyper-concentration, increased viscosity and subsequently to an impaired mucus clearance and persistent neutrophil airway inflammation and bacterial colonization [6]. Therefore, subjects with CB experience more exacerbations, an increased risk of accelerated decline in lung function and decreased survival [7].

The major macromolecular components of the mucus in human airways are the mucin glycoproteins MUC5B and MUC5AC [8–10]. In the healthy lung, MUC5AC is mainly produced by epithelial surface goblet cells found in both large and small airways, whereas MUC5B is predominantly secreted from mucus cells in submucosal glands of the large trachea and the bronchial tubes [10, 11]. However, in patients with inflammatory airway diseases including COPD, MUC5B is also upregulated in airway epithelium in both large and small airways [12–14]. Thus, the pathological changes in the airway epithelia lead to persistent elevated secretion of mucus from epithelial goblet cells into the respiratory tract, including distal airways [15].

In COPD/CB, macrophages are believed to play a major role in orchestrating the inflammatory response [16]. Together with epithelial cells and the mucus layer, they constitute the first line of defense against infections and noxious agents. Although the number of alveolar macrophages is increased in bronchoalveolar lavage (BAL) from healthy smokers [17], and from both current and ex-smoking COPD patients [18], a vast body of evidence suggest that these cells are functionally impaired with reduced ability to release pro-inflammatory cytokines in response to bacterial lipopolysaccharides (LPS) [19–22]. In contrast to these findings, the levels of macrophage-derived pro-inflammatory mediators such as TNF-α, CCL3 and IL1β are reported to be strongly increased in sputum from both current and ex-smoking COPD patients [2]. This apparent discrepancy suggests specific chronic activation of macrophages in sputum rather than a general chronic activation of lung macrophages.

Neutrophilia is a key feature of chronic bronchitis and a complex network of cytokines and chemokines including IL-8 (CXCL8), CXCL1/5/7 and CCL3 are known chemotactic factors for neutrophil migration to inflammatory sites through binding to CXCR1/2 and CCR1/5 receptors [23–25]. Further, inflammasome-activated IL1β has been shown to drive neutrophil influx through an IL-8 independent mechanism in vivo [26] and in vitro exposure to TNFα has been shown to increase the ability of human neutrophils to migrate towards CCL3 through CCR5-mediated activity [27]. Thus, TNFα, CCL3 and IL1β, which are found in high concentrations in sputum, can all influence neutrophil recruitment as well as sustain the inflammatory process in neutrophilic lung disease.

Even though the clinical manifestations of CB are well characterized, little is known about the underlying signaling driving sputum hyper-production and the contribution of different cell types to the clinically observed corticosteroid resistance in this chronic inflammatory disease. We hypothesized that the highly neutrophilic and pro-inflammatory cytokine milieu in sputum plugs may drive development of a steroid-resistant, chronically activated macrophage phenotype that constantly secretes inflammatory signals driving mucus hyper-production by epithelial cells.

In this study, we aimed to investigate the nature of macrophage-epithelial signaling driving mucus hyper-production using a newly established mechanistic in vitro model and to compare the LPS and steroid responsiveness of clinically sampled macrophages isolated from sputum plugs from patients with COPD and chronic bronchitis to that of alveolar macrophages isolated from lung resection tissue. Some of the results of these studies have been previously reported in the form of abstracts [28, 29].

## Materials and methods

### Sampling of sputum macrophages

#### Study design and patients

The current analysis was part of an exploratory study of inflammatory responses in blood and cells isolated from induced sputum from COPD/CB patients, an observational single-center study. This study was conducted in accordance with the amended Declaration of Helsinki and approved by the ethics committee at the University of Gothenburg, Sweden, Approval number 501-17 and T 820-17. Written informed consent was obtained from all patients.

Patients with CB, defined as a history of cough with sputum expectoration for at least 3 months a year during a period of 2 consecutive years, ascertained by oral interview and questionnaires were included in the study. Patients with COPD (GOLD I-III) aged 60–73 years, in a clinically stable state, were prospectively recruited between September 2017 and March 2018 at the COPD Centre at Sahlgrenska University Hospital, Sweden. Patients currently on PDE4 inhibitors or systemic glucocorticosteroids were excluded from the study.

Sixteen patients were recruited into the study and all were successful in producing induced sputum. However, there were only 9 patients, where we were able to isolate sufficient numbers of viable sputum macrophages to study LPS and/or steroid responsiveness in technical triplicates (> 0.8 × 10^6^ cells), that were included in the current analyses (Table 1).

**Table 1:** Subject characteristics of sputum donors

Clinical data								Ex vivo assays			
Patient	Age span	Pack years	FEV1 (L)	FEV1% of pred	FEV1/FVC (%)	CAT^c^	CB-Q^d^	qPCR *TNF*	qPCR *IL1B*	qPCR *CCL3*	Steroid resistance
1	70–74	50	2.6 (3.1^a^)	59 (71^a^)	53 (59^a^)	22	34	X	X		
2	70–74	7	2.4	79	69	16	31	X	X		
3	70–74	44	1.8	57	61	29	38	X	X		
4	60–64	47	1.0 (1.1^a^)	39 (44^a^)	43 (47^a^)	31	42	X	X	X	
5	70–74	75	2.5	88	52	31	39	X	X	X	X
6	60–64	8^b^	4.3 (4.3^a^)	97 (98)^a^	61 (63^a^)	19	31				X
7	65–69	33	1.7 (2.2^a^)	44 (56)^a^	46 (51^a^)	16	27	X	X	X	X
8	65–69	47	1.5 (1.4^a^)	60 (57^a^)	62 (64^a^)	35	39	X	X	X	X
9	65–69	46	1.4 (1.6^a^)	56 (63)^a^	45 (45^a^)	9	28	X	X	X	X

^a^Spirometry with bronchodilation

^b^Pipe

^c^COPD assessment test

^d^Chronic bronchitis questionnaire

#### Assessments

All patients underwent physical examination, including demographic information, medication history and smoking status at the inclusion visit. CAT (COPD assessment test) was performed [30] and a short CB questionnaire (Additional file 1: Table S1) to assure active CB phenotype. All patients performed spirometry (IntraMed, LaunchSentrySuite) and a pulmonary x-ray.

At the second visit, within 3 weeks after the inclusion visit, induced sputum and blood samples were collected. Five millilitre of peripheral venous blood was sampled for analyses of white blood cell differential tests and high-sensitive C-reactive protein (CRP) at the Department of Clinical Chemistry, Sahlgrenska University Hospital (Table 2).

**Table 2:** Inflammatory markers in blood

Patient	CRP (mg/l)	Leukocytes (× 10^9^/l)	Neutrophils (× 10^9^/l)	Eosinophils (× 10^9^/l)	Lymphocytes (× 10^9^/l)
1	1.4	9.3	2.8	0.2	5.7
2	N/A^a^	7.9	5.3	0.1	2.0
3	6.0	7.7	4.9	0.6	1.4
4	3.2	7.1	3.7	0.1	2.9
5	4.5	7.6	4.8	0.1	2.0
6	1.0	7.9	5.1	0.4	1.8
7	17	10	6.6	0.2	2.6
8	1.9	6.8	4.9	0.2	1.1
9	1.2	10	6.4	0.1	3.0

^a^Not available

#### Sputum induction

Sputum induction through inhalation of hypertonic NaCl was performed using a standardized protocol [31] with some minor adjustments. Prior to induction, the baseline FEV1 (forced expiratory volume in 1 s) was measured and patients with FEV1 < 40% of predicted capacity were excluded to reduce the risk of adverse events. Patients were pre-medicated with inhaled Salbutamol 400 µg and FEV1 was again measured. The nebulisation started with 3% hypertonic saline solution with an inhalation of 7 min (tidal breathing) followed by coughing and expectoration to harvest sputum samples. If the subject was not able to produce sputum the procedure was repeated using increased hypertonic saline solutions (4% and 5% NaCl). FEV1 was measured after every round of inhalation. If FEV1 fell more than 20% from the post-salbutamol value or if the subject developed symptoms, the procedure was stopped. We used the Hedenström values, applying a non-linear age coefficient for lung function decline, as references for spirometry [32, 33]. Sputum samples were put on ice and processed within 1 h.

### Sampling of alveolar macrophages

#### Study design and patients

Alveolar macrophages (AM), were isolated from human lung resections from 7 patients with COPD and 10 patients without COPD, undergoing lung cancer resection surgery at the Department of Cardiothoracic Surgery at Sahlgrenska University Hospital. Clinical characteristics are presented in Additional file 2: Table S2. Samples were obtained in accordance with hospital and AstraZeneca ethical guidelines with written consent from all patients. Ethics committee Approval number 1026-15.

### Studies on sputum and alveolar macrophages

#### Sputum macrophage isolation and cell culture

Sputum macrophages (SM) were isolated from human induced sputum plugs by 5–10 min incubation in 4 volumes (4 × weight of plugs, final DTT concentration = 0.08%) Sputolysin (Millipore) on an Intelli-mixer RM2S (ELMI) rotating vortex mixer (program F8 at 20 rpm) [31]. The sputum/Sputolysin mixture was inspected after 5 min and every minute thereafter to immediately proceed with the protocol as soon as the viscosity of the mixture changed drastically. The mixture was diluted to 0.04% DTT with cold PBS and filtered through a 70 µM cell strainer, centrifuged at 300*g*, 4 °C for 5 min and the sputum cell pellet was subsequently washed 3 times in 5 ml cold PBS. A small portion of the sputum cells was transferred to glass slides by cytospin and stained with May–Grünwald Giemsa for differential counting (Table 3). More than 400 non-squamous cells from each sample were scored by two independent researchers and squamous cell count was < 10% in all samples, 1.8% (0.2–5.3), median (range). The sputum cell pellets were resuspended in warm Xvivo10 (Lonza #BE04-743Q) supplemented with 2 mM l-glutamine, 1× penicillin–streptomycin, 1.25 μg/ml Amphotericin B (all from Gibco, Life technologies) and seeded at a density of 1 × 10^5^ cells/well in 96 well tissue culture treated microplates (Costar #3595, Corning Inc.). The macrophage fraction was purified by attachment to cell culture plastics for 1 h, followed by three repeated washes with 100 µl warm Xvivo10 media to remove non-attaching cells such as neutrophils, eosinophils and lymphocytes. Treatment with 0.1% DMSO (Merck) or 50 nM dexamethasone (Calbiochem) was performed overnight at 37 °C and 5% CO_2_. After overnight resting or compound or DMSO control treatment, the cells were either stimulated for 2 h (RNA isolation) or 6 h (measurement of soluble TNFα in media) with 100 ng/ml LPS (*E. coli*, clone 0127:B8, Sigma #L4516). Macrophage viability, monitored in replicate wells at the end of the in vitro assays, was 83% (81–88), median (range). The purity of the culture after purification of macrophages by attachment was monitored by differential staining during the setup of the method (> 90%, n = 3). During the study, the purity of the washed culture was monitored by visual inspection of cell morphology. A similar protocol for adherence purification of sputum macrophages was recently published by Bolling et al. [34].

**Table 3:** Inflammatory cells in sputum

Patient	Sputum cell composition (%)^a^				Counts (10^6^)^a^
	Macrophages	Neutrophils	Eosinophils	Lymphocytes	Total cells
1	26.2	66.0	4.0	3.8	4.4
2	12.2	82.3	1.5	3.9	9.8
3	22.8	73.1	1.0	3.1	6.5
4	14.1	83.2	1.2	1.5	7.6
5	4.1	94.4	0.2	1.3	23.8
6	10.0	63.7	25.4	0.9	10.7
7	22.1	74.0	2.7	1.2	9.2
8	9.8	86.9	0.5	2.8	10.2
9	23.1	74.5	0.6	1.8	4.4

^a^Non-squamous cells. Squamous cell count < 10% in all samples, 1.8% (0.2–5.3), median (range)

#### Alveolar macrophage isolation and cell culture

AM were isolated by repeated flushing of lung resection tissue with PBS using a 19G needle. The collected flush was pelleted by centrifugation 300*g*, 5 min. AM pellets were resuspended in Xvivo10 (Lonza #BE04-743Q) supplemented with 2 mM l-glutamine, 1× penicillin–streptomycin, 1.25 μg/ml Amphotericin B (all from Gibco, Life technologies) and seeded at a density of 1 × 10^6^ total cells/ml in tissue culture treated 25 cm^2^ flasks (Nunc #156367, Thermo Scientific). The macrophage fraction was purified by attachment to cell culture plastics for 1 h, followed by three repeated washes with 5 ml Xvivo10 media. Cultures were rested, or treated with 50 nM Dexamethasone or DMSO, overnight before stimulation with 100 ng/ml LPS for 2 h (RNA isolation), 6 h (measurement of soluble TNFα in media) or 24 h (conditioned media) at 37 °C and 5% CO_2_. Cell viability prior to seeding were 92% (90–96), median (range). The purity of the macrophage culture after purification by attachment was monitored by differential staining during the setup of the method (> 95%, n = 3). During the study, the purity of the washed cultures was monitored by visual inspection of cell morphology.

#### RNA isolation and quantitative RT-PCR

Total RNA was extracted using RNeasy micro kit (#74034, Qiagen) and reverse transcription was performed using the ABI high capacity cDNA kit (#4368813, Life Technologies) according to the manufacturer’s instructions. RT-qPCR was carried out using TaqMan gene expression assays (*TNF* Hs00174128_m1, *CCL3* Hs00234142_m1, *IL1Β* Hs01555410_m1, *GUSB* Hs00939627_m1 and *POLR2A* Hs00172187_m1) on the ABI Quantstudio 7 Flex Real-Time PCR System. Data are normalized to reference genes (*GUSB* and *POLR2A*) and presented as fold change (dCT) or as log2 mean fold change relative to basal conditions (ddCT).

#### Detection of soluble TNFα

The Meso Scale Discovery electrochemiluminescence assay (MSD, Gaithersburg) was used to measure absolute levels of TNFα in cell free supernatants from macrophage cultures according to the manufacturer’s protocol.

#### Alveolar macrophage—human bronchial epithelial cell cross-talk assay

Cell numbers isolated from clinical sputum samples were too low (~ 0.2–2 × 10^6^ cells/subject) to generate sufficient amounts of conditioned media for the macrophage-epithelial cross-talk assay. For this reason, cell-free conditioned media from alveolar macrophages, which can be isolated in vast numbers from lung resections, were used to study macrophage-HBEC cross-talk in this mechanistic in vitro study.

To generate pseudo-stratified air–liquid-interface (ALI) epithelial cell cultures [35], normal human bronchial epithelial cells (HBECs) (donor #0000448571 and #0000485960, Lonza) at passage 2, were seeded at 6.3 × 10^4^ cells/cm^2^ on 6.5 mm polyester insert Transwell plates (#3478 with #3395, Corning). The cells were submerged in complete PneumaCult-Ex Plus media (#05040, STEMCELL Technologies) until air-lift at day 4 after seeding. The cells were then grown in ALI culture in complete PneumaCult-ALI media (#05001, STEMCELL Technologies) with media renewal every 2–3 days. After the initiation of mucus production, at approximately day 14 after air-lift, apical mucus was washed off every 2–3 days by incubating the apical cell surfaces with complete media for 2–3 h at 37 °C 5% CO_2_ before restoration of ALI. At day 21, the sufficiently differentiated cultures were washed, and the basolateral media was replaced by fresh ALI-media, 1:1 ALI-media + LPS-supplemented (100 ng/ml) Xvivo10 media, 1:1 ALI-media + LPS activated macrophage-conditioned Xvivo10 media with or without the addition of 1 µg/ml neutralizing anti-TNFα monoclonal antibody (clone cA2, #RAB00046, Abnova) or by 50 ng/ml huTNFα (#210-TA/CF, R&D Systems) in ALI-media with or without 1 ug/ml neutralizing anti-TNFα monoclonal antibody. Triplicate wells/treatment were stimulated for 48 h at 37 °C, 5% CO_2_. The apical cell surfaces were then washed with 100 μl PBS (Ca^2+^ and Mg^2+^ free) for 1 h at 37 °C. The cells were fixed with 4% formaldehyde (#9713100, VWR) for 20 min at RT, washed three times with PBS and stored submerged in PBS at + 4 °C for immunocytochemistry.

#### Immuno-cytochemistry of whole-mount ALI cultures

The fixed cells were washed once with PBS and permeabilized with PBS + 0.25% TritonX-100 (#1001541166, Sigma-Aldrich) for 20 min. The inserts were washed three times and blocked with PBS + 1% BSA (#1002119388, Sigma Aldrich) + 2% goat serum (#S-1000, Vector labs) at + 4 °C overnight, then incubated with PBS + 1% BSA + 2% goat serum + 5 µg/ml anti-MUC5B (#ab77995, Abcam) for 2 h. The cells were washed four times for 5 min and stained with PBS + goat anti-mouse IgG Alexa Fluor568 (#A21124, Invitrogen, 1:400) + Alexa Fluor 488 Phalloidin (#A12379, Invitrogen, 1:50) for 1 h, protected from light. The inserts were washed two times for 5 min and counterstained with PBS + Hoechst 33342 (#62249, Thermo Fisher Scientific, 1 µg/ml) for 10 min. After four 5-min washes, the membranes were removed from the culture inserts and mounted using Vectashield mounting media (#H-1000, Vector labs). Three representative images/membrane were captured using an LSM 880 Airyscan microscope (Zeiss) and the percentage of MUC5B/total image area was quantified using the Visiopharm Integrator System (Visiopharm A/S). All immunocytochemistry data are presented as mean of three independent experiments using cell-free conditioned media from three different AM donors and triplicate technical replicates.

#### Proteome profiler analysis of ALI cultures

ALI-HBEC, stimulated basolaterally for 30 min, with 1:1 ALI-media + Xvivo10 media with 100 ng/ml LPS or 1:1 ALI-media + LPS-activated macrophage-conditioned media at day 21 after air-lift, were processed and analyzed according to the manufacturer’s instructions in the proteome profiler human phospho-kinase array kit (ARY003B, R&D Systems). Mean pixel densities were calculated from scanned X-ray films using Image Studio 4.0 (LI-COR Biosciences).

### Statistical analysis

Experimental data are presented as individual experiments with mean ± standard deviation or range when indicated. Clinical data are presented as median with (range). For single comparisons, one-sided Welch t-tests were used and for multiple comparisons, one-way ANOVA with Šidák’s correction for multiple comparisons were performed. QQ plots were used to check for normality assumption and a sandwich estimator was used to obtain a consistent estimate of the variance of the residuals due to heteroscedasticity. Analysis was performed in R [36] and data was plotted using GraphPad Prism 8. Significant p-values are represented as *p < 0.05; **p < 0.01; ***p < 0.001; ns = not significant.

## Results

### Subject characteristics

Clinical characteristics of the chronic bronchitis COPD cohort at time of inclusion are presented in Tables 1, 2 and 3. All patients were current or former tobacco smokers with smoking pack years history of 41.8 (8–75), median (range), and had a diagnosis of chronic bronchitis and COPD GOLD stage I, II or III.

Median of COPD assessment test (CAT) was 22 (9–35) out of a maximum value of 40 and median of the short CB questionnaire 34.3 (27–42) out of a maximum value of 45 confirming active disease of CB in this cohort.

Blood test showed that median of CRP 2.55 (1.0–17) mmol/l [reference value < 5 mmol/l], leukocytes 7.9 (6.8–10) [3.5–8.8] * 10^9^/l and neutrophils 4.9 (2.8–6.6) * 10^9^/l [1.8–7.5] were all in the upper part of reference values. On the contrary, median of eosinophils 0.2 (0.05–0.6) [0.04–0.4] * 10^9^/l, was in the lower part of reference values.

Further, as expected in this patient population, sputum cell counts showed an increase in sputum neutrophils 77.6 (63.7–94.4)% (Table 3) compared to 69% that has been reported for age-matched healthy volunteers [37]. Taken together, these data indicate that the patients tested here correlate well with a neutrophilic COPD phenotype.

### Human bronchial epithelial cells grown in ALI culture are inert to direct LPS stimulus but respond when the inflammatory signal is relayed via macrophages

**Fig. 1:**
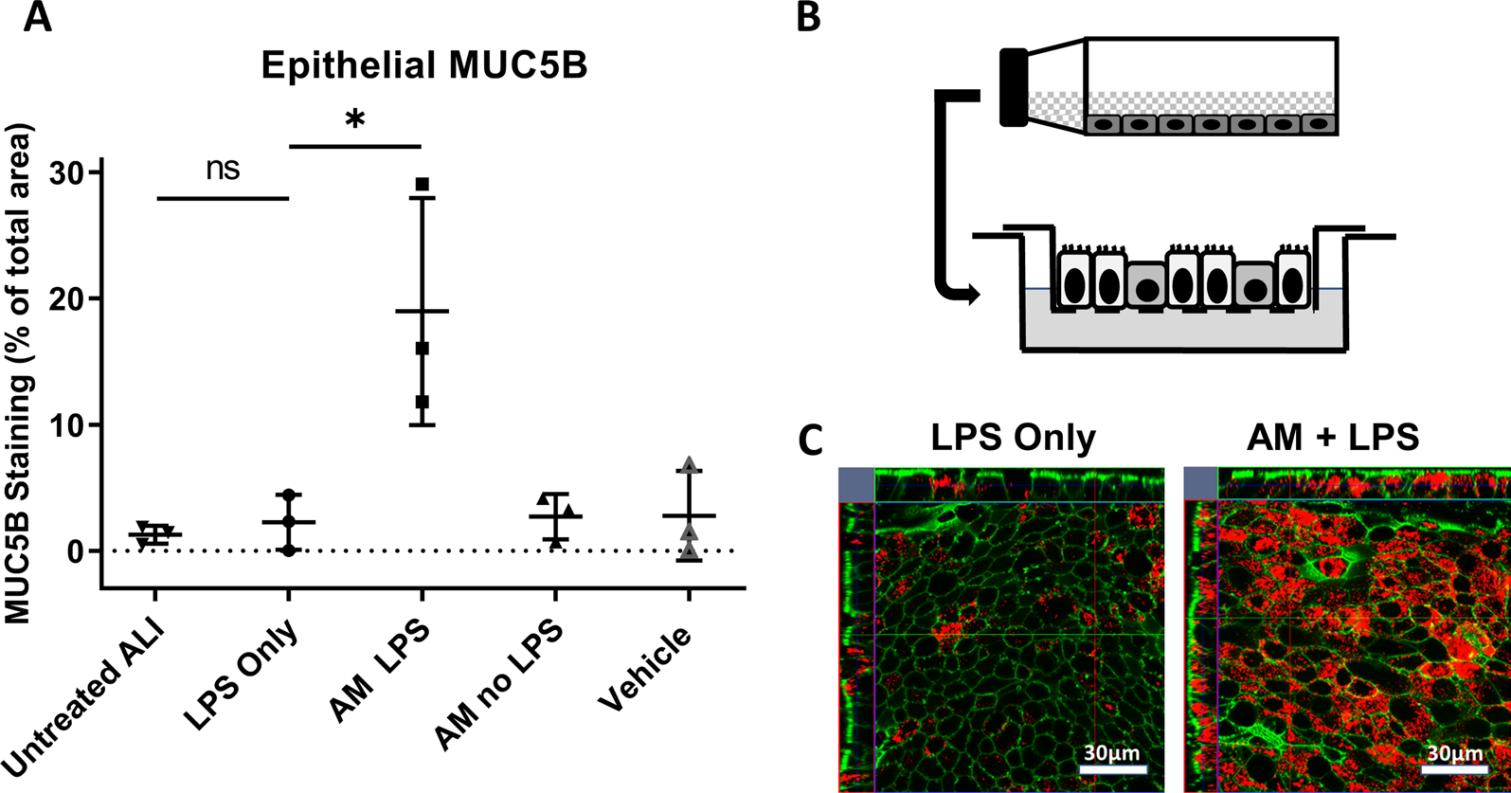
Macrophages relay inflammatory signals to epithelial cells through a soluble factor. Intracellular MUC5B staining of human bronchial epithelial cells (HBEC), grown in ALI culture. **A** Five different conditions were investigated in HBECs: Untreated ALI, media with 100 ng/ml LPS, conditioned media from LPS activated AM from three different donors, conditioned media from same AM donors without LPS or by addition of 50% Xvivo-10 macrophage media alone (Vehicle control). Data are presented as mean ± standard deviation from three independent experiments using conditioned media from three different AM donors and triplicate technical replicates. **B** Schematic drawing depicting basolateral transfer of conditioned macrophage media to ALI cultures in transwell plates. **C** Representative confocal scans of MUC5B in whole-mount ALI cultures stimulated with LPS only or conditioned media from LPS activated macrophages (Green = Phalloidin, Red = MUC5B)

The effect of LPS stimulation on primary human bronchial epithelial cells (HBEC) grown as ALI cultures was investigated by measuring the production of the major mucus component, MUC5B (Fig. 1A). Direct basal stimulation of HBEC ALI cultures by LPS (LPS only) caused no increase in MUC5B expression (p = 0.97). In contrast, the conditioned media from LPS stimulated AMs (AM LPS) evoked a significant increase in MUC5B expression in the ALI cultures (p = 0.017). In line with this finding, neither conditioned media from unstimulated AM (AM no LPS), nor media alone (Vehicle) had any effect on epithelial MUC5B expression. Taken together, these data clearly show that the inflammatory signal is relayed through the alveolar macrophages and mediated by a soluble factor, which was later investigated (see below).

### Conditioned media from LPS-activated alveolar macrophages phosphorylate CREB/AP1 transcription factors in human bronchial epithelial cells

**Fig. 2:**
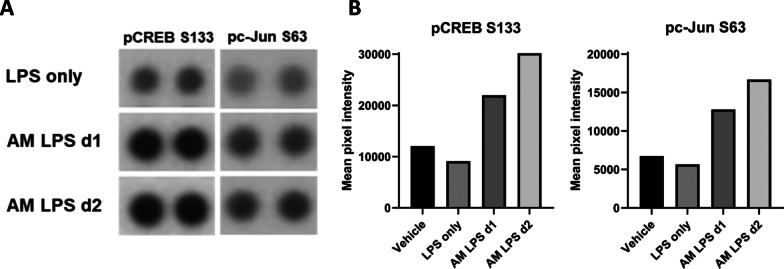
Macrophage signaling lead to activation of CREB and AP-1 transcription factors. **A** Phosphorylation of CREB S133 and c-Jun S63 sites in primary human bronchial epithelial cells grown in ALI culture after 30 min of basolateral stimulation with vehicle media, LPS only or conditioned media from LPS activated alveolar macrophages from two lung resection tissue donors (d1, d2). **B** Quantification of mean pixel intensities from duplicate spots in each donor

To unravel the signaling pathways leading to increased MUC5B expression after addition of conditioned media from LPS stimulated alveolar macrophages, we performed a kinase phosphorylation analysis in ALI cultured HBECs including 43 different human kinases (Proteome profiler array). After 30 min of stimulation by conditioned media from activated alveolar macrophages from two different AM donors (d1 and d2), the kinases CREB and c-Jun showed increased phosphorylation on S133 and S63, respectively, compared to basic stimulation with LPS alone (Fig. 2). These data suggest that macrophage signaling lead to activation of CREB and AP-1 transcription factor complexes which are both considered key players regulating mucin genes in human epithelial cells [38, 39].

### Macrophage-derived TNFα is a key driver of MUC5B in bronchial epithelial cells

**Fig. 3:**
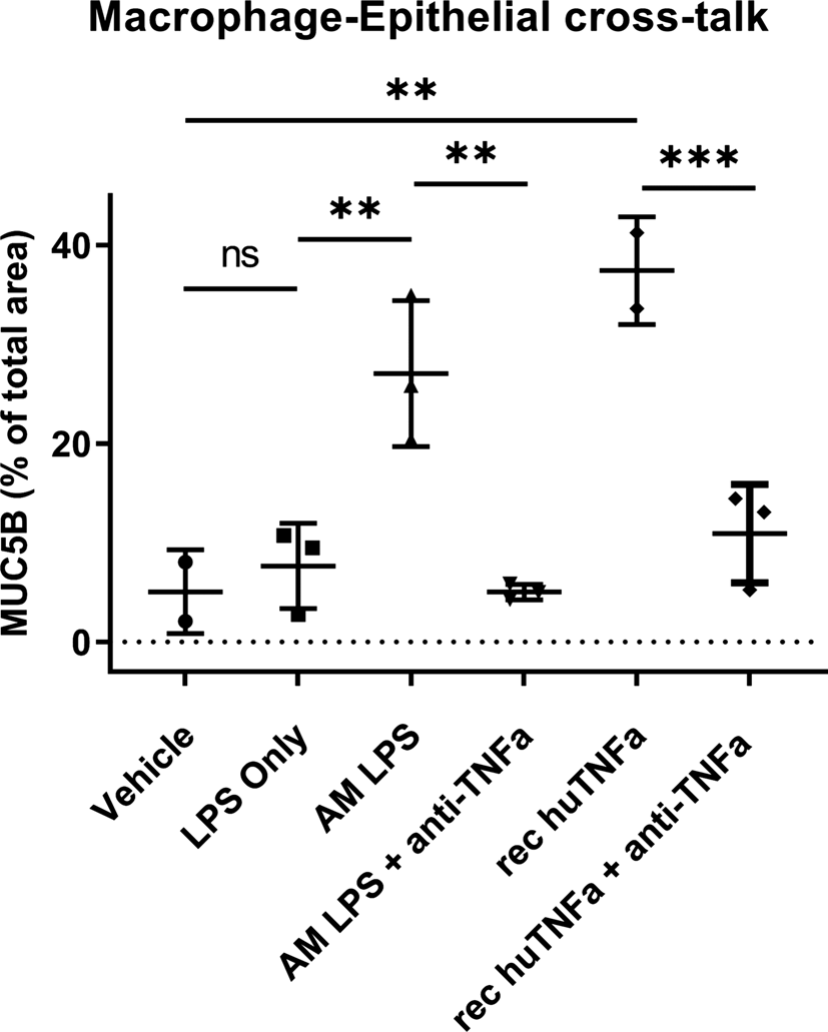
TNFα released from macrophages in response to bacterial LPS, plays a key role in driving mucus production in bronchial epithelial cells. Intracellular MUC5B staining in whole-mounts of human bronchial epithelial cells, grown as ALI cultures. Conditions include: Unstimulated (Vehicle), direct stimulation with LPS, stimulation with conditioned media from LPS activated macrophages w/wo addition of a TNFα-neutralizing antibody or direct stimulation with 50 ng recombinant TNFα w/wo addition of a TNFα-neutralizing antibody**.** Data are presented as mean ± standard deviation from three independent experiments using conditioned media from three different AM donors and triplicate technical replicates

The major LPS inducible macrophage-derived mediator TNFα has been shown to activate CREB and AP-1 transcription factor complexes through TNFR1 (via p38MAPK/MSK1) and TNFR2 (via JNK) [40, 41]. We next investigated the possible involvement of TNFα in mediating the downstream effects on MUC5B expression in epithelial cells grown in ALI culture using neutralizing antibodies and recombinant human TNFα (Fig. 3). Direct stimulation of the epithelial cells by recombinant human TNFα (rec huTNFa) resulted in a strong upregulation of MUC5B (p = 0.0003). Again, conditioned media from activated macrophages (AM LPS) resulted in a significant upregulation of epithelial MUC5B (p = 0.0035). Interestingly, the addition of neutralizing TNFα antibodies to the conditioned media from LPS stimulated alveolar macrophages (AM LPS + anti-TNFa) completely abolished the effect of macrophage-conditioned media on epithelial MUC5B expression (p = 0.0014). Also, addition of neutralizing TNFα antibodies to the recombinant TNFα media (rec huTNFa + anti-TNFa) reduced the expression of MUC5B to basal levels (p = 0.0008). Taken together, these mechanistic data show that TNFα released from macrophages in response to bacterial LPS, plays a key role in mucus production in bronchial epithelial cells.

### Macrophages from chronic bronchitis sputum are chronically activated

**Fig. 4:**
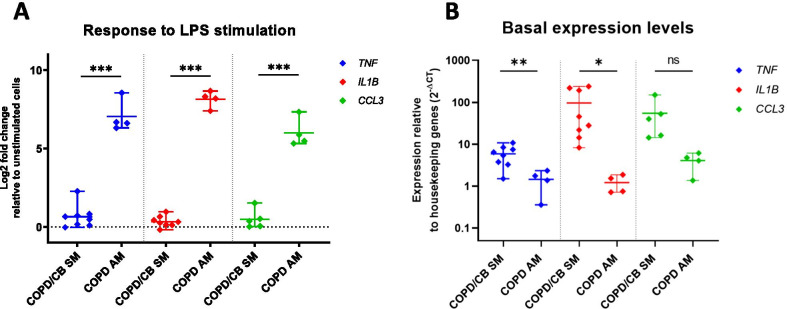
Macrophages from chronic bronchitis sputum are chronically activated and respond poorly to further LPS stimulation. Effect on pro-inflammatory gene expression by LPS (100 ng/ml) stimulation of sputum (SM) and alveolar macrophages (AM). **A** Log2 fold-change vs unstimulated cells (ddCT) and **B** showing basal RNA expression levels as 2^−dCT^ compared to the house-keeping genes *POLR2A and GUSB*. Data are presented as mean ± range

Expression of the pro-inflammatory genes encoding *TNF*, *IL1B* and *CCL3* by 2 h LPS stimulation was studied in ex vivo isolated sputum macrophages from CB patients and compared to the effect seen in alveolar macrophages isolated from lung resections. After LPS stimulation, no significant fold-change (ddCT) induction of RNA levels of these genes were seen in sputum macrophages, showing that these cells are unresponsive to LPS stimulation (Fig. 4A). However, by comparing levels of basal *TNF*, *IL1B* and *CCL3* RNA expression normalized against the *POLR2A* and *GUSB* housekeeping genes (dCT), we showed that TNFa (p = 0.0041) and IL1B (p = 0.033) transcripts were already partially increased in unstimulated sputum macrophages showing that this cell population is chronically activated in the lung environment (Fig. 4B). In contrast to the effects seen in sputum macrophages, alveolar macrophages isolated from lung resections show little or no basal activation but are strongly activated in vitro by LPS stimulation (p = 0.00008; p = 0.00001; p = 0.00007 for *TNF*, *IL1B* and *CCL3* respectively) (Fig. 4A).

### Sputum macrophages from COPD chronic bronchitis patients are steroid resistant

**Fig. 5:**
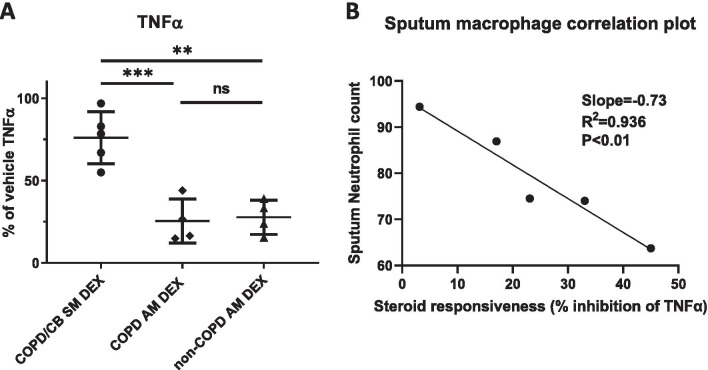
Sputum macrophages from COPD chronic bronchitis patients are steroid resistant. **A** Effect of 50 nM dexamethasone (DEX) on TNFα protein levels in media from LPS stimulated CB sputum macrophages (COPD/CB SM) (n = 5) and alveolar macrophages (AM) from COPD or non-COPD patients (n = 4). Cells were pre-treated with compound or DMSO vehicle for 22 h and subsequently stimulated with LPS (100 ng/ml) for 6 h. Data are presented as mean ± standard deviation. **B** Linear regression correlation plot comparing sputum neutrophil differential counts (%) and sputum macrophage steroid responsiveness (% inhibition of TNFα at 50 nM dexamethasone)

Given the importance of TNFα as mediator in the mechanistic macrophage-epithelial cross-talk assay and the chronic *TNF* expression observed in the clinically sampled sputum macrophages, the effect of the corticosteroid dexamethasone was investigated by measuring the amount of TNFα protein released into the media of ex vivo cultured sputum and alveolar macrophages. When exposed to dexamethasone (50 nM) prior to LPS stimulation, only partial reduction in TNFα levels was seen in sputum macrophages (24% vs vehicle, n = 5) isolated from chronic bronchitis COPD patients (COPD/CB SM DEX), compared to a significantly larger response in both COPD alveolar macrophages (75% vs vehicle, n = 4), (COPD AM DEX) as well as non-COPD alveolar macrophages (72% vs vehicle, n = 4), (non-COPD AM DEX) isolated from lung resections (Fig. 5A). Taken together, these data show that, in contrast to alveolar macrophages from either COPD or non-COPD donors, sputum macrophages from COPD chronic bronchitis patients are steroid resistant (p = 0.0008 and 0.0011, respectively). Further, comparison of the sputum neutrophil differential counts and steroid responsiveness (% inhibition of TNFa) revealed a potential direct correlation between sputum neutrophils and sputum macrophage steroid resistance (R^2^ = 0.936, p = 0.0071) (Fig. 5B).

## Discussion

In the current study, we have evaluated the inflammatory state of purified sputum macrophages from COPD chronic bronchitis patients and for the first time investigated the response to pharmacological inhibition by steroids in this isolated cell fraction. We found an apparent disconnect in both the inflammatory state and in the response to glucocorticoids between macrophages isolated from sputum or alveolar macrophages isolated from lung resections, suggesting chronic activation and steroid resistance in sputum-derived macrophages. Further, using a primary human macrophage and bronchial-epithelial cell co-culture system we show that LPS signaling at the epithelial surface is relayed through macrophages and that macrophage-derived TNFα is a key mediator activating CREB/AP1-driven MUC5B expression in lung epithelial cells.

Chronic bronchitis is associated with persistent airway inflammation in the lining of bronchial tubes and an increase in mucus production leading to mucus hyper-concentration and bacterial colonization driving further mucus production. The hyper-concentration of mucus positively correlates with exacerbation frequencies and has been proposed as a disease driver in chronic bronchitis with MUC5B remaining the predominant mucin at all levels of disease severity [13, 42]. Epithelial cells are relatively inert to LPS stimulation and previous reports have shown that only very high LPS concentration over a long period can drive mucus expression in experimental systems of lung epithelium [43]. On the other hand, macrophages isolated from lung resections, readily respond to even low amounts of LPS. Thus, macrophages seem to function as sentinels of the lung by sensing the presence of pathogens and respond by cytokine/chemokine signaling. Cross-talk between macrophages and lung epithelium may be instrumental in providing optimal host protection against infections and noxious agents as the mucus layer provides an efficient barrier to trap and remove bacteria by the mucociliary escalator system. However, in disease this system is perturbated, resulting in chronic stimulation and activation of signals driving mucus hyper-production and ultimately hyper-concentration, thus impairing mucociliary clearance and leaving the cough reflex as the essential remaining functional clearance mechanism. Clinically, this is presented as the productive sputum and cough defining a chronic bronchitis patient [4].

We have set up a novel, fully primary human cell system to study macrophage-epithelial cross-talk driving MUC5B protein expression. For practical reasons, alveolar macrophages were used to perform these mechanistic studies, investigating how signals released from macrophages can drive mucus expression in epithelial cells. Firstly, AM can be isolated in large quantities by flushing of resection tissue. Secondly, they are quiescent cells and release very low or undetectable levels of TNFα when cultured in vitro without stimuli and they are also highly responsive to LPS stimulation, which was confirmed by us at the RNA level before and after LPS stimulation (Fig. 4). Using this system, we clearly show that bacterial LPS can activate the alveolar macrophage, which in turn signals through the release of TNFα to the epithelial cells. This signal, in turn activates MUC5B protein production in the epithelial columnar goblet cells that secrete gel-forming mucins. In this respect, the ALI culture of pseudostratified human bronchial epithelia represents a good model of COPD/CB airway epithelium exhibiting goblet cell meta/hyperplasia [8]. Further, using a phospho-proteome profiler approach we show that macrophage signaling activates CREB S133 and c-Jun S63 in epithelial cells supporting previous reports of CREB and AP-1 regulation as key transcription factors regulating mucin genes in human epithelial cells [38, 39]. TNFα has the potential to activate both these transcription factors in lung epithelial cells, similar to what has previously been shown in endothelial cells where TNFα activates CREB and c-JUN through TNFR1 (via p38MAPK/MSK1) and TNFR2 (via JNK), respectively [40, 41]. Interestingly, both IL1β and TNFα can activate CREB S133 phosphorylation in human nasal epithelial cells [44] and the MUC5B promoter harbors numerous transcription factor sites for CREB [45]. Other stimuli such as reactive oxygen species (ROS) emanating from cigarette smoke exposure have been shown to directly activate the MUC5B defense mechanism in epithelial airway cells [12]. While both activation of macrophages and direct activation of epithelial cells by ROS are indeed possible drivers of disease during the development of chronic bronchitis, the chronic activation of SM reported here, may represent a mechanism behind persistent mucus secretion perpetuating the disease state even after smoking cessation.

Despite the accessibility of sputum macrophages, ex vivo pharmacological intervention of inflammatory responses in purified populations of these cells has not previously been investigated. However, studies of acute effects on total sputum cells confirm steroid resistance and LPS unresponsiveness which may be a consequence of the unique environment these cells are residing in, both regarding cytokine and chemokine levels, but also considering the ROS and proteases released by the vast number of neutrophils in this niche [46].

Recently, a method describing the isolation and culturing of human sputum macrophages from healthy volunteers was published, with similarities to the method described here [34]. In this paper, the authors showed that even though sputum macrophages exhibited a highly pro-inflammatory signal directly after isolation, the levels were reduced after 20 h of culturing and that the ex vivo macrophages responded with lower but significant TNFα expression in response to stimulation with LPS. The difference between this report and the lack of LPS responsiveness in our study may be explained by differences in culture conditions, LPS dose (1 µg/ml compared to the 100 ng/ml used in our study) or by the origin of the macrophages since our study used selected sputum plugs while this report used the entire expectorate as source for sputum macrophage isolation. By comparison to alveolar macrophages from lung resections, we clearly show that there is a large difference in response to LPS between sputum macrophages (< twofold in our study and ~ threefold in the study by Bolling et al. [34]) and alveolar macrophages (~ 100-fold in this study), suggesting that statistically relevant induction and biologically relevant induction of TNFα may differ. Previous investigations in healthy subjects have shown that there is a clear difference, both in functional activation [47] and metabolic activity [48], between sputum and alveolar macrophages and it is likely that the findings described here is a consequence of distinct local microenvironments present in the upper and lower respiratory tract rather than a direct feature of chronic bronchitis. However, in COPD chronic bronchitis patients, the numbers of sputum plugs, neutrophils and macrophages are increased as a consequence of the chronic inflammatory state of their lungs, which may perpetuate disease through the vicious cycle of sputum hyper-production and hyper-concentration. In fact, it was recently shown that mucus plugs and emphysema was independently associated with FEV1 and that the effect sizes on airflow obstruction between these entities were similar [49]. Further, sputum neutrophilia correlated with high mucus plug scores, high COPD Assessment Test (CAT) scores and more frequent annual exacerbations [49], showing the importance of mucus plug abundance in disease progression.

Several investigations have shown high pro-inflammatory cytokine and chemokine levels in sputum supernatants including TNFα, IL1β and CCL3 [2, 50]. While this may appear counter-intuitive to the lack of response to LPS found in this study, it may be explained by a moderate, chronic expression over time. Chronic activation of macrophages makes them less efficient to evoke a strong immediate peak in pro-inflammatory cytokine expression and this may reduce their ability to counteract bacteria entering the lung and could possibly represent a basis for bacterial colonization of the lung, frequently seen in COPD patients.

In this study, we show that purified sputum macrophages from patients with chronic bronchitis and COPD exposed to dexamethasone, show steroid resistance compared to alveolar macrophages isolated from non-COPD and COPD patient lung resections, thus identifying a possible basis for the clinically observed steroid resistance in the COPD/CB patient population.

Contrary to the situation in asthmatic patients, inhaled steroids in COPD have been shown to be much less effective in improving lung function and controlling the underlying chronic inflammation. Only a fraction of COPD patients show a direct clinical response to inhaled corticosteroids and these patients often have increased numbers of eosinophils and show bronchodilator reversibility [51]. However, maintenance steroid treatment is still part of the current clinical practice to reduce exacerbation frequency in COPD/CB. Several mechanisms and mediators are known to be implicated in the development of steroid resistance. The neutrophilic inflammation generates local oxidative stress that activates lung epithelial cells and alveolar macrophages, from which pro-inflammatory cytokines are released. These inflammatory cytokines are potent mediators for steroid resistance by suppressing the glucocorticoid receptor [52]. Further, inflammasome activation and subsequent IL1β expression, known to be present in COPD, has been shown to drive experimental severe, steroid-resistant asthma [53]. In this context, it is interesting to note that the sputum macrophages showing the most pronounced resistance to the effect of dexamethasone were derived from the subject with the highest percentage of neutrophils in sputum plugs (subject 5), while the sputum macrophages from the patient with the least neutrophils and instead the highest eosinophil percentage in sputum plugs (subject 6, possibly a mixed COPD/Asthma phenotype) shows the least steroid resistance of all patients (Fig. 5B). While these data suggest a direct correlation between sputum neutrophil content and steroid resistance, replication of these data in a larger cohort is needed to fully establish this connection. TNFα-neutralizing antibodies have shown good effects in multiple auto-immune chronic inflammatory condition including rheumatoid arthritis, Crohn’s disease and psoriasis [54]. However, despite multiple clinical trials to evaluate anti-TNFα agents in COPD patients, no clear efficacy could be established warranting use in this disease [55, 56]. The failure of anti-TNF-α therapy in COPD may have several causes. If, in coming studies, the phenotypes of COPD would be better characterized, one could more specifically identify which patients may benefit from TNFα inhibitors [57]. However, since many, if not all, of the current trials lack target engagement markers confirming the ability of the agent to reach the target TNFα producing cells, it would be of particular interest to study access of anti-TNFα antibodies or antibody-like proteins into sputum plugs where we suggest the main fraction of steroid resistant cells reside in COPD.

The main limitation of the current study is the small number of COPD/CB patients studied, the restricted numbers of sputum macrophages that could be isolated from each patient and the lack of a control group of either non-bronchitis COPD patients or healthy subjects that would have enabled a disease vs non-disease comparison. However, given the large differences in baseline proinflammatory gene expression and response to LPS and corticosteroids observed between SM and AM, this study was still powered to detect significant phenotypic hallmarks of CB sputum macrophages, but further studies with larger sample sizes are needed to more accurately quantify the differences described here. Similarly, restricted numbers of isolated SM from induced sputum samples prevented dose–response evaluation of dexamethasone in these cells. To circumvent this limitation, a dose–response investigation in AM was performed (data not shown), enabling us to select a precise dose to detect differences between SM and AM. Also, comparison of SM which are considered a heterogenous population regarding morphology, maturation and activation state to the more homogenous AM popolation may complicate statistical comparison due to differences in the distribution of data. However, statistical methods monitoring normality assumption and variance estimates were used to assure proper statistical comparison between these groups.

In conclusion, our data pinpoint a critical role for sputum macrophages in chronic bronchitis by perpetuating macrophage-epithelial signaling that can drive aberrant mucin expression in bronchial epithelial cells. Novel strategies targeting the steroid-resistant, chronically activated phenotype of sputum plug macrophages are warranted to drive resolution of chronic inflammation in the lungs of patients suffering from COPD with chronic bronchitis.

## Supplementary Information

**Additional file 1: Table S1.** CB questionnaire.

**Additional file 2: Table S2.** Subject characteristics of lung resection tissue donors.

## Data Availability

All data used in this manuscript are provided within the Main manuscript or in the Additional data files.

## Abbreviations

COPD: Chronic obstructive pulmonary disease
CB: Chronic bronchitis
AM: Alveolar macrophage
SM: Sputum macrophage
BAL: Bronchoalveolar lavage
LPS: Lipopolysaccharide
TNF-α: Tumor necrosis factor-α
IL: Interleukin
CCL: Chemokine (C–C motif) ligand
CXCR: CXC chemokine receptors
CCR: C–C chemokine receptor
PDE: Phosphodiesterase
FEV1: Forced expiratory volume in 1 s
CAT: COPD assessment test
CRP: C-reactive protein
ALI: Air–liquid-interface
HBEC: Human bronchial epithelial cell
