# Supplementary material for: Lung macrophages drive mucus production and steroid-resistant inflammation in chronic bronchitis
Source: Respir Res. 2021 Jun 7;22:172. doi: 10.1186/s12931-021-01762-4 (PMC8186034; doi:10.1186/s12931-021-01762-4)
Supplement: Supplementary file 1 — Additional file 1: Table S1. CB questionnaire. [file 12931_2021_1762_MOESM1_ESM.docx]

**Table S1. CB Questionnaire.**

1. My cough never hurts 0 1 2 3 4 5 My cough always hurts
2. My cough does not affect me 0 1 2 3 4 5 My cough affects me considerably
3. My phlegm (sputum) doesn’t affect my breathing 0 1 2 3 4 5 My phlegm (sputum) makes it hard to breathe
4. I never bring up phlegm (sputum) 0 1 2 3 4 5 I bring up phlegm (sputum) daily
5. It is easy to cough up phlegm (sputum) 0 1 2 3 4 5 I am having troubles to cough up phlegm (sputum)
6. My phlegm (sputum) is not thick at all 0 1 2 3 4 5 My phlegm (sputum) is very thick
7. My phlegm (sputum) does not bother me 0 1 2 3 4 5 I am seriously bothered by my phlegm (sputum)
8. The last 4 weeks I have coughed:

1. Not at all

2. Only when I had an infection in the lung

3. A couple of days per month

4. Several days a week

5. Almost every day

9. The last 4 weeks I have brought up phlegm (sputum):

1. Not at all

2. Only when I had an infection in the lung

3. A couple of days per month

4. Several days a week

5. Almost every day
