# Supplementary material for: Lung macrophages drive mucus production and steroid-resistant inflammation in chronic bronchitis
Source: Respir Res. 2021 Jun 7;22:172. doi: 10.1186/s12931-021-01762-4 (PMC8186034; doi:10.1186/s12931-021-01762-4)
Supplement: Supplementary file 2 — Additional file 2: Table S2. Subject characteristics of lung resection tissue donors. [file 12931_2021_1762_MOESM2_ESM.pdf]

Table S2. Subject characteristics of lung resection tissue donors.

Clinical data								*in vitro* assays			
**Donor**	**Age span**	**Smoking status**	**Pack years**	**Diagnosis**	**FEV1 (L)**	**FEV1 (% of pred.)**	**FEV1 /FVC (%)**	**Proteome profiler assay**	**Cross-talk assay**	**LPS response**	**Steroid resistance**
1	70-74	XS	N/A	PE*	3.1	104	71	X	X		
2	65-69	XS	19	PC‡	2.1	94	68	X	X		
3	80-84	NS	0	TP§	2.7	121	73		X		
4	70-74	XS	15	TP§	3.6	104	73		X		
5	70-74	XS	25	TP§	1.1	50	58		X		
6	75-79	XS	N/A**	TP§	1.7	78	65		X		
7	75-79	XS	N/A**	LC†/COPD	1.2	52	53			X	
8	80-84	XS	40	LC†/COPD	2.3	70	69			X	
9	45-49	XS	30	LC†/COPD	2.9	85	67			X	
10	70-74	XS	2	LC†/COPD	1.5	70	69			X	X
11	75-79	XS	N/A**	TP§	2.4	115	77				X
12	80-84	NS	0	TP§	2.5	99	64				X
13	55-59	NS	0	HDTL||	N/A**	N/A**	N/A**				X
14	75-79	NS	0	TP§	N/A**	N/A**	N/A**				X
15	85-89	NS	0	LC†/COPD	1.8	72	69				X
16	75-79	XS	8	LC†/COPD	1.9	73	69				X
17	80-84	XS	N/A**	LC†/COPD	1.5	78	51				X

*Pulmonary Embolism, †Lungcancer, ‡Precarinal Tumor , §Tumor Pulmonae., ||Heathy donor transplant lung, **Not available.
